# A Novel Technique to Prevent Femoral Artery Occlusion With Impella CP Sheaths: Case Report

**DOI:** 10.1016/j.jscai.2025.103871

**Published:** 2025-09-09

**Authors:** Kristina Khaw, Said Ashraf, Howard Levite, Jeffrey Van Hook, Kenneth Khaw

**Affiliations:** a Cooper Medical School of Rowan University, Camden, New Jersey; b AtlantiCare Regional Medical Center, Pomona, New Jersey

**Keywords:** case report, impella, large-bore access, limb ischemia prevention, ventricular assist device

## Abstract

Impella CP use in small femoral arteries has increased vascular and ischemic complications. Various techniques have been described to lessen leg ischemia. Our technique uses a small catheter placed below the Impella CP insertion site and runs parallel with it to prevent total occlusion in the femoral artery. This could be a primary technique to try first. If more distal flow to the leg is needed, antegrade access to the superficial femoral artery can be obtained with connection to the adjacent wedge catheter. We describe this use for first time in a patient with cardiogenic shock after myocardial infarction with mitral regurgitation.

## Introduction

The Impella CP left ventricular assist device (Abiomed) is often used in high-risk coronary artery revascularization and in cardiogenic shock associated with acute myocardial infarction. Sometimes, the Impella CP is left in the femoral artery (FA) for continued left ventricle support after the procedure; however, patients with a small FA often do not get the Impella CP, as its large sheath would occlude the FA. Various approaches have been used to prevent leg ischemia. One such approach is to make holes in the Impella sheath.^1^ Another approach is to obtain antegrade access into the FA^2^ with the blood supply coming from the contra- or ipsilateral^3^ FA or radial artery.^4^ Our wedge technique is simple and quick. First, a sheath is placed below where the Impella CP sheath (ImpCPSh) would be accessed. This physical wedge makes a space between the ImpCPSh and FA to prevent occlusion. This can avoid the leg ischemia seen in 3% to 5%^5^^,^^6^ of patients receiving an Impella CP.

This wedging technique was first used on a 64-year-old obese female patient (82.3 kg, 1.6 m, body mass index 33.1 kg/m^2^) who presented to the emergency department with an acute inferolateral myocardial infarction with complete heart block. A temporary transvenous pacer was placed in the internal jugular vein. She was emergently brought to the catheterization laboratory on maximum dosages of epinephrine and norepinephrine. Ultrasound was used to access both the FA and the right femoral vein. An angiogram showed a small nondominant right coronary artery, left anterior descending artery with 85% proximal tandem stenoses, and occlusion of a very large dominant circumflex artery. She had a 3.0 × 18 mm stent in the proximal circumflex artery and a 2.0 × 22 mm stent in the first obtuse marginal artery. On the left ventriculogram, moderate mitral regurgitation was seen with hyperkinetic anterior wall and an ejection fraction of 35% to 40%. A 40-mL intra-aortic balloon pump was placed in the right FA. Right heart catheterization showed mean right atrial pressure of 24 mm Hg, pulmonary artery pressure of 62/42 mm Hg (mean 49), and pulmonary wedge pressure of 45/54 mm Hg (mean 45). Fick cardiac output was 6.5 L/min and index 3.7. She was started on dual antiplatelet therapy and transferred to the cardiac unit. Her condition deteriorated the next morning. The balloon pump was exchanged with a vascular sheath. The left FA was accessed according to the method described below. Wedging and ImpCPSh were placed without any complication. The peel-away sheath was removed after angiogram. The Impella CP remained in the patient for 11 days without leg ischemia.

## Method description

1. A 5F micropuncture needle was used to access the common FA just above the profunda artery (Figure 1). Then, a 4F catheter (Pinnacle, Terumo Medical Corporation) was placed.

**Figure 1:**
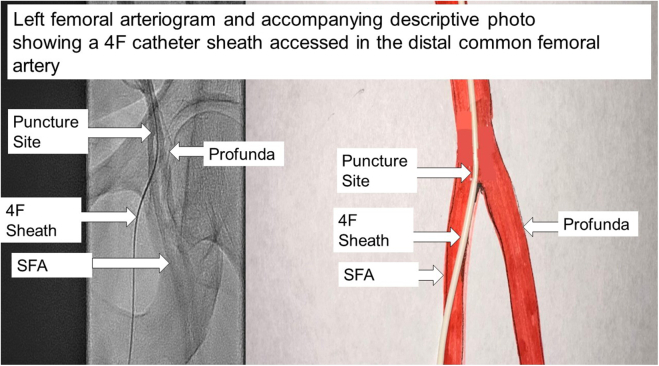
**Left femoral arteriogram with a 4F catheter sheath accessed above the profunda artery for wedging catheter.** SFA, superficial femoral artery.
2. A quick angiogram was performed through the catheter to help obtain access for the ImpCPSh at the mid common FA (Figure 2).

**Figure 2:**
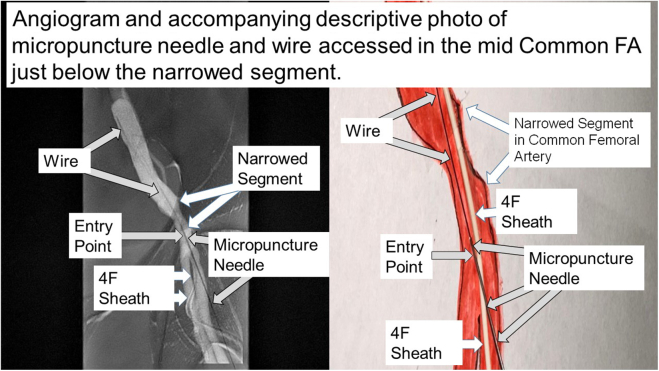
**Micropuncture needle and wire entered the mid common femoral artery just below the narrowing using digital subtraction image.** FA, femoral artery.
3. The Impella CP peel-away sheath was inserted over the wedging sheath.
4. An angiogram (Figure 3) was performed through the wedge sheath to check for adequate flow in the FA distal to the ImpCPSh. If more flow is needed, a larger sheath can be used. The figure shows blood was able pass around the ImpCPSh (large parallel linear lines at distal iliac artery) at the narrow FA area.

**Figure 3:**
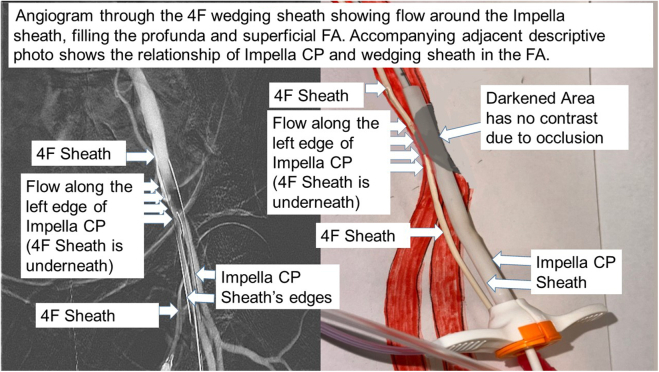
**Angiogram through the 4F wedging sheath showing flow around the Impella sheath, filling the profunda and superficial femoral artery.** FA, femoral artery.

To show that blood can flow around the wedging catheter, a bench setup was made with heat-shrink tubing (modeled as the FA) encompassing the ImpCPSh and the wedging sheath. The tubing was shrunk down to maximally compress the wedge sheath (20 cm length) with the ImpCPSh. The setup was attached to a saline fluid source at 100 mm Hg pressure on 1 side. The model was tested using different sizes: 4F, 5F, and 6F sheaths (Pinnacle). Flow rates measured were 38, 52, and 64 mL/min, respectively. These are not brisk flows; however, flow rates can be significantly higher when the occlusion wedge length with the ImpCPSh becomes shortened (Figure 3). If this length is 5 cm instead of 20 cm, flow rates can be 4 times higher.

This simple catheter wedge technique allowed blood to flow around the ImpCPSh to prevent total occlusion. This should be considered as a primary technique to prevent leg ischemia. If there is not enough distal flow, an antegrade catheter can be placed in the superficial FA and connected to the wedge sheath. This will allow more Impella CP to be used in small FA vessels.
